# The Combination of RNA and Protein Profiling Reveals the Response to Nitrogen Depletion in *Thalassiosira pseudonana*

**DOI:** 10.1038/s41598-017-09546-x

**Published:** 2017-08-21

**Authors:** Jianbo Jian, Dezhi Zeng, Wei Wei, Hongmin Lin, Ping Li, Wenhua Liu

**Affiliations:** Marine Biology Institute, Shantou University, Shantou, Guangdong, 515063 P.R. China

## Abstract

Nitrogen (N) is essential for the growth of algae, and its concentration varies greatly in the ocean, which has been regarded as a limitation for phytoplankton growth. Despite its great importance, most of the existing studies on the mechanisms underlying the effects of N on diatoms have focused on physiology, biochemistry and a few target genes and have rarely involved whole genomic analyses. Therefore, in this study, we integrated physiological data with RNA and protein profiling data to reveal the response strategy of *Thalassiosira pseudonana* under N-depleted conditions. Physiological measurements indicated that the cell growth capacity and chlorophyll content of the cells decreased, as did the expression of photosynthesis- and chlorophyll biosynthesis-related genes or proteins. The RNA-Seq profile results showed that *T. pseudonana* responded to N deprivation through increases in glycolysis, the TCA cycle and N metabolism as well as down-regulation in the Calvin cycle, gluconeogenesis, pentose phosphate, oxidative phosphorylation and lipid synthesis. These results provide a basic understanding for further research addressing how N affects phytoplankton in terms of genomics.

## Introduction

As an essential element in living creatures, nitrogen (N) plays a vital role in the composition of macromolecules and in determining the intracellular proportions of key metabolites of N and carbon metabolism^1^. The N content of the coastal zones has increased for decades because of the human nitrogen economy in agriculture^2^. However, compared with terrestrial plants, nitrogen is still a major limiting nutrient for primary production in the ocean^3^. Therefore, N is often used as a stressor treatment to study physiological changes in microalgae^3–6^.

Due to the great contribution of diatoms to global primary production^7^, *Thalassiosira pseudonana* is one marine diatom that is widely distributed throughout the world’s oceans and is often used as a model for diatom physiology or lipid-accumulation studies^8, 9^. Because of its wide distribution and small genome size, *T. pseudonana* was also the first eukaryotic marine phytoplankton whose whole genome sequence was decoded^10^. In the past few decades, most research has focused on physiological and biochemical phenomena, such as responses to nitrate, ammonium, silicon, phosphorus, light, temperature and CO_2_ in algae^11–15^, whereas only a few studies have attempted to examine whole transcripts or changes at the protein level^5, 16–18^. In fact, the algal genome era has become a hot topic in recent years, and an increasing number of algae genomes have been completed, including those of *Cyanidioschyzon merolae* ^19^, *Ostreococcus tauri* ^20^, *Phaeodactylum tricornutum* ^21^ and *Porphyridium purpureum* ^22^.

There are have been many studies on relevant stressors based on microarray analyses, 2-dimensional electrophoresis and transcriptome and proteome methods^5, 9, 23^. A recent study revealed whole-cell alterations stimulated by phosphorus deficiency by analyzing both the global transcriptome and proteome^24^. Additionally, there have been studies addressing the effects of iron, silicon and other macronutrient stressors on *P. tricornutum* ^17, 25–27^. In the diatom *T. pseudonana*, several previous research studies have focused on gene expression responses related to different environment stress conditions using the available technology. According to transcript analysis performed through RT-PCR, the effects of phosphine provide an alternative mechanism to oxidation to release phosphate for uptake^9^; 16 copper-induced genes were identified under copper stress^27^; and the expression levels of multiple selective genes were studied under nitrogen, phosphate and iron depletion stress conditions^28^. Using microarray methods, the dose-response effects of the polycyclic aromatic hydrocarbon benzo[a]pyrene on diatoms were detected^29^, and a wealth of new genes were identified as potentially being involved in silica formation in response to silicon stress^8^. Compared with the methods used in these studies, high-throughput sequencing or proteome methods, or de novo transcript expression can generate much more information. A total of 318 transcripts or 136 proteins in multiple biochemical pathways were found to be differentially abundant under diverse forms of phosphorus stress^24^, and large-scale proteome and transcriptome rearrangement was shown to be induced by cobalamin scarcity^18^.

Under very severe N-stress, the extent for the N-stress altering cell metabolism and growth depends on how severe the stress is. The traditional research has shown *T. pseudonana* undergoes little or no additional cell division, and its uptake capacity declines under deeply N-depleted conditions^11^. There is also a reduction of the intracellular chlorophyll *a* (Chl *a*) content, which is responsible for the decreasing light absorption^30^, and an increase in pigment and dimethyl sulfoniopropionate contents^12, 15^. Numerous studies have shown that N depletion may lead to lipid accumulation (mainly of triacylglycerol, or TAG) in numerous microalgal species, such as *Chlamydomonas reinhardtii*, *Nannochloris sp*., *Scenedesmus obliquus*, *Parietochloris incise*, and *Haematococcus pluvialis* ^31–37^. As a model species with one of the earliest available reference genomes for eukaryote algae, the N-stress response of *C. reinhardtii* is well understood^36, 38, 39^. In *C. reinhardtii*, transcript analysis suggested that N depletion can directly lead to the funneling of acetate into fatty acid biosynthesis, causing a significant increase in lipid droplets, whereas protein biosynthesis is down-regulated, and a subset of control genes involved in gametogenesis are activated^38^. Moreover, rapidly developing post-genomics biological approaches have become practical for obtaining an in-depth understanding of how microbes respond and adapt to variations in the external environment, especially among algae without genome sequences. There are also a few studies based on RNA-seq methods that have focused on the molecular changes in non-sequenced microalgae when N is limited in culture conditions, especially in the biosynthesis pathway responsible for lipid formation and accumulation^34, 40–45^.

To investigate the response and alterations of *T. pseudonana* metabolic pathways induced by N depletion, we combined physiological data, RNA-Seq analysis and proteomic profiling to analyze diverse responses through mapping using the reference genome in the Joint Genome Institute (JGI) database. The results of this analysis can help us to further understand how this microalga fundamentally responds to physiological N stress. Both RNA-seq and proteome data were used in comparisons and to identify positional polymorphisms as well as differential expression levels. Through integrated analysis of RNA-seq and proteome profiles, we obtained differentially expressed transcripts and proteins as well as correlated items, which were further analyzed through functional annotation and pathway enrichment analyses. In this study, we used Nile Red dye to stain cells to semi-quantify the lipid content in cells for monitoring of intracellular secondary metabolic conditions. When systematic calculation was performed, 782 genes and 362 proteins showing differential expression between the treatments emerged. Our goal was to gain thorough insight into the molecular changes that promote or accompany lipid accumulation in *T. pseudonana* cells, especially for genes associated with photosynthesis, carbon fixation and N uptake, and we aimed to gain a whole-cell view of adaption and survival to further explain some of the physiological phenomena that are observed in N-depleted environments.

## Materials and Methods

### Culture conditions

A Chinese *T. pseudonana* strain was cultivated at 20 ± 1 °C under 220 μmol of photons m^−2^ s^−1^ and a 12 h photoperiod. Algal cells were initially activated twice in complete f/2 medium, and sodium nitrate was the only N supplement. The cells were transferred when they reached the exponential stage, at a cell density of approximately 5.5 × 10^4^ cells/ml, and were then cultured under N-replete (NR) (f/2 medium, with an initial 882 μM nitrate concentration) or N-depleted (ND) (f/2 medium minus nitrate, approximately 182 μM initial nitrate concentration) conditions; the day of the transfer was set as day 1. Cell growth conditions were monitored daily, including measurements of cell density, chlorophyll content, Nile Red fluorescence and free N (nitrate and nitrite) remaining in the medium. To estimate chlorophyll contents, 20 ml of cultivated cells were harvested using GF/C filters (Whatman, UK), after which the filters were immersed in 6 ml of 90% acetone. Following incubation of the cells at 4 °C for at least 16 h in darkness, the filters and cells were removed via centrifugation, and the supernatant fluid was subjected to OD measurements using an Infinite M200 Pro microplate reader (Tecan, Switzerland), with 90% acetone as the blank (Mitchell, BG, 1984). The chlorophyll content was estimated using the following formulas^46^:

$${\rm{Chl}}\,{a}={\rm{11}}{{\rm{.47OD}}}_{{\rm{664}}}-{\rm{0}}{{\rm{.40OD}}}_{{\rm{630}}}$$

$${\rm{Chl}}\,{c}_{1}+{c}_{2}=24.36O{D}_{630}-3.73O{D}_{664}$$

To quantify lipid levels, 1 ml samples of the cultures were mixed with Nile Red solution (0.1 mg/ml in acetone) (100/1, v/v) and were then analyzed after 7 min on an Infinite M200Pro microplate reader (Tecan, Switzerland). The fluorescence emission spectra were measured at 580 nm, with 544 nm as the excitation wavelength^47^. The N concentration remaining in the medium was determined from measurements of nitrate and nitrite concentrations^48^. All conditions were assessed in triplicate. The cells were harvested in mid-stationary phase using sterile 0.45 μm filters, and all samples were snap-frozen in liquid nitrogen and stored in a −80 °C freezer.

### RNA extraction and RNA-Seq data analysis

Total RNA was obtained from the snap-frozen N-replete and N-deprived (day 4) cultures described above using the TRIzol reagent (Invitrogen, USA) according the manufacturer’s protocol. First, the cells were treated with DNase I to remove DNA contamination, and the total RNA was then enriched using oligo(dT) magnetic beads. The procedures for library construction and sequencing were consistent with previous reports^49^. Briefly, the purified mRNA was fragmented into ~200 bp fragments that were suitable for sequencing. After the first strand of cDNA was successfully synthesized using random hexamer primers, the second strand was synthesized. The double-stranded cDNA was again purified with magnetic beads. After end reparation and ligation of the sequence adaptors, the modified fragments were amplified via PCR. During the QC step, an Agilent 2100 Bioanalyzer (USA) and an ABI StepOnePlus Real-Time PCR System (USA) were used to qualify and quantify the sample libraries. Finally, the library products were sequenced with an Illumina HiSeq^TM^ 2000 system (USA).

For base calling, the raw image data were converted to sequence data with a length of 49 bp, which were defined as the raw data. The adaptor sequences were first removed, and the raw reads were processed by filtering out low-quality data (more than 10% unknown bases; more than 50% of bases showing Q 5 within a read). The retained high-quality reads, which were considered clean reads, were then mapped to reference sequences and reference genes (http://genome.jgi-psf.org/Thaps3/Thaps3.home.html) using SOAP2^50^, allowing 3 and 2 mismatches, respectively, in the alignment. The methods for identifying differentially expressed genes (DEGs) were the same as the methods used in a previous study^51^ based on the Poisson distribution model. An “FDR (false discovery rate) ≤0.001 and an absolute value of the log_2_Ratio ≥1 (fold-change ≥2)” were used as the threshold to judge the significance of differences gene expression^52^. Cluster 3.0^53^ software was subsequently used to perform cluster analysis of gene expression patterns.

### Isolation and preparation of total protein

Both samples were ground to a powder in liquid nitrogen. Total protein was then extracted according the manufacturer’s recommendations in the next step. Briefly, the samples were mixed with lysis buffer (7 M urea, 2 M thiourea, 4% CHAPS, 40 mM Tris-HCl pH 8.5, 1 M PMSF and 2 mM EDTA). After adding 10 mM DTT, the suspension was sonicated and centrifuged. Then, the supernatant was mixed with a 5-fold volume of chilled acetone, followed by incubation at −20 °C for 2 hr. The resultant protein pellets were suspended in lysis buffer, then sonicated and centrifuged again. To reduce disulfide bonds, the supernatant was incubated at 56 °C with 10 mM DTT for 1 hr. Subsequently, the samples were incubated in darkness for 45 min with 55 mM IAM added to block the cysteines. The samples were then pelleted using chilled acetone and reconstituted in 0.5 M TEAB (Applied Biosystems). Finally, the supernatant was transferred to a new tube and quantified using a Bradford Protein Assay Kit. Next, 100 μg of total protein was taken from each sample solution and digested with Trypsin Gold (Promega, USA) into peptides. According the manufacturer’s protocol for the 8-plex iTRAQ reagent (Applied Biosystems, USA), the peptides were labeled with the isobaric tag and dried via vacuum centrifugation. SCX fractionation was performed with an LC-20AB HPLC Pump system (Shimadzu, Japan), and the peptides were pooled into 20 fractions, which were subsequently loaded into an LC-20AD nanoHPLC (Shimadzu, Japan). Data acquisition was performed with a TripleTOF 5600 System (AB SCIEX, USA) fitted with a Nanospray III source (AB SCIEX, USA) and a pulled quartz tip as the emitter (New Objectives, USA).

### Proteome analysis

The proteome analysis included peptides identification, protein identification and protein quantification. The mass tolerance of 0.05 Da (ppm) was permitted for intact peptide masses, allowing for one missed cleavage in the trypsin digests. To reduce the probability of false peptide identification, only peptides showing significance scores ≥20 in the 99% confidence interval in a Mascot probability analysis as falling above the “identify” threshold was counted as identified peptides. Then, using Mascot 2.3.02 (Matrix Science, London, United Kingdom)^54^, mass spectrum data was converted to MGF (Mascot generic format) format and the final protein identification results were obtained against a database containing *T. pseudonana* sequences. In this process, every confidently identified protein involved at least two unique peptides and FDR for the peptide spectrum level was set as < = 0.01. The detailed procedures are described in http://www.matrixscience.com/help/quant_statistics_help.html. With the Mascot 2.3.02 software, the student’s T-test was performed in this analysis. Briefly, a protein ratio is reported in bold face if it is significantly different from unity. The comparison test is:

$$|\bar{x}-\mu |\le t\,\ast \,\frac{s}{\sqrt{N}}$$

There is no significant difference at the stated confidence level when this inequality is true. (N is the number of peptide ratios, *s* represents the standard deviation and *x* are the mean of the peptide ratios, both numbers calculated in log space. The true value of the ratio, µ, is 0 in log space. *t* is student’s t for *N*-1 degrees of freedom and a two-sided confidence level of 95%). Finally, the protein quantification and statistical significance of the protein quantitative ratios was performed by the software of Iquant^55^. The ratio between the NR and the ND was obtained directly using the protein abundance for any given protein. We used p-value < 0.05 and only those with fold changes >1.5 were considered as differentially expressed proteins. In IQuant analysis, the mascot percolator algorithm methods was combined^56^ and protein-level FDR < = 0.01 was used to decrease the false positive rate^57^. The statistical analysis refers to permutation tests which have been widely used in the fields of microarray and RNA-Seq data analysis^58, 59^.

### Functional annotation

For both the RNA-Seq and proteome data, functional annotations were conducted using the BLAST2GO (Gene Ontology) program, along with the non-redundant protein database (Blastnr; NCBI). The cluster of Orthologous Groups (COG) database (http://www.ncbi.nlm.nih.gov/COG/) was employed to classify and group the putative and definitively identified proteins. The KEGG database (http://www.genome.jp/kegg/) was used to obtain maps of regulated metabolic pathways in molecular interaction and reaction networks.

## Results and Discussion

### Physiological performance during N deprivation

**Figure 1:**
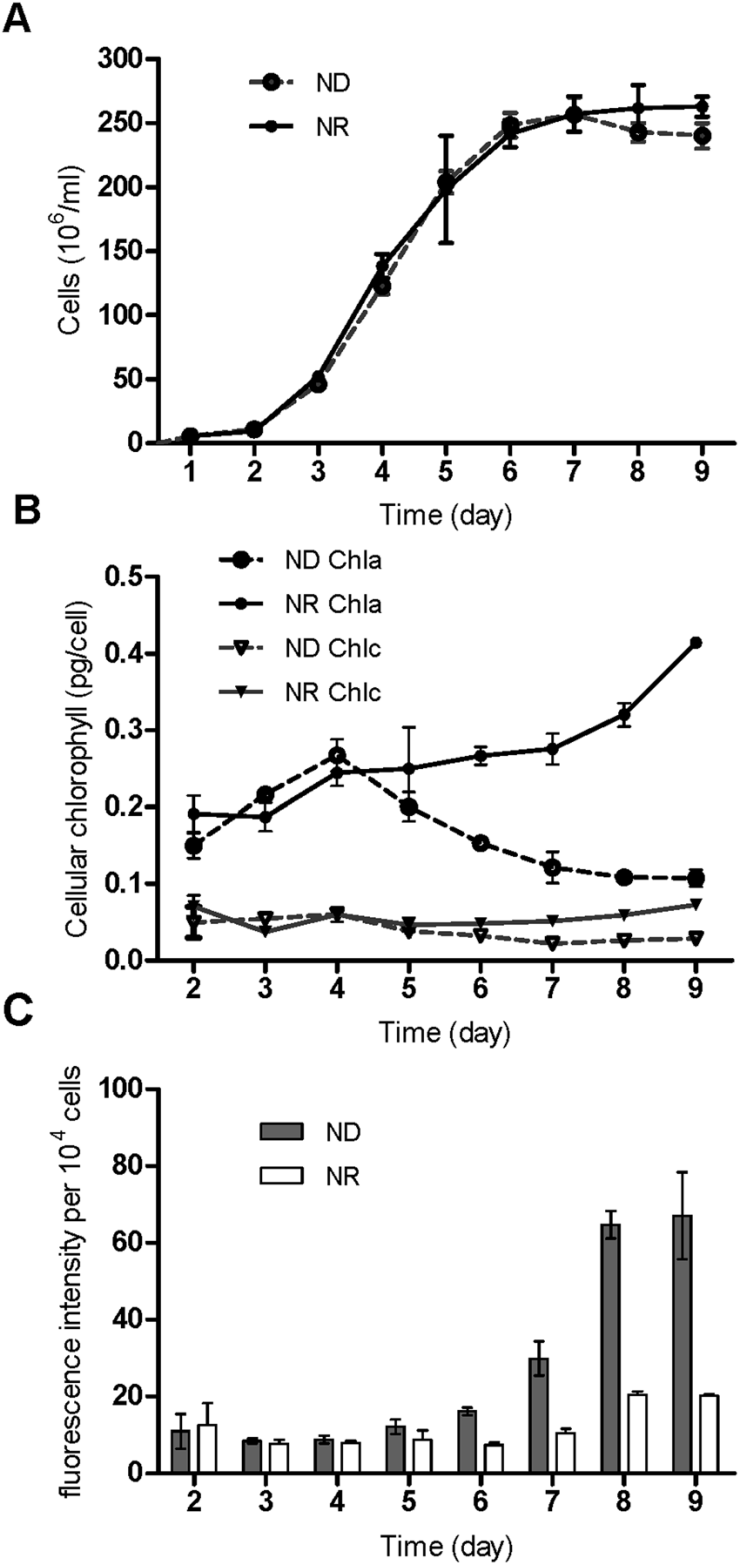
Physiological parameters of *T. pseudonana* under different N levels. The entire cultivation period was 9 days. (**A**) Growth of algal cells under N-replete (NR) and N deprivation (ND) conditions. (**B**) *T. pseudonana* cellular chlorophyll content in NR and ND treatments. (**C**) Nile Red fluorescence intensity per 10^4^ cells. The data are displayed as the means ± SD (n = 3).

To monitor their growth, the *T. pseudonana* cultures were sampled at the same time (12:00) every day and were used to measure cell counts, chlorophyll contents, Nile Red fluorescence and the free N remaining in the medium. Through the examined period, the N deprivation treatment showed a similar growth curve to N repletion and the cell counts obtained under both conditions increased from an initial count of 5.57 × 10^4^ cells/mL to a plateau of 2.57 × 10^6^ cells/mL on day 7. Then, the count under N deprivation slightly declined to 2.40 × 10^6^ cells/mL (Fig. 1A). Different N concentrations also led to other substantial physiological changes. As observed under a microscope, algae cell size was smaller under the N deprivation condition (data not shown). The contents of Chl *a* and Chl *c*_*1*_ + *c*_*2*_ showed a continuous increase overall in f/2 medium, with Chl *a* increased from 0.15 to 0.27 pg/cell at the early stage (before 96 h). On day 4, a decrease in the chlorophyll a content was observed (Fig. 1B). Similar results were observed during nitrogen depletion in *Phaeodactylum tricornutum and Neochloris oleoabundans* ^60, 61^. The fluorescence intensity value obtained under N deprivation was 3.4-fold of that under N repletion, indicating that the neutral lipid content was significantly higher on day 8 (Fig. 1C). Regarding the N content in the culture environment, the trend in changing nitrate levels was like that for Chl *a*. The nitrate concentration showed a continuous drop and even became exhausted under the N deprivation condition (Figure S1A,B). Results showed that the nitrate and nitrite were indeed consumed in the ND treatment, but there was always remained nitrate in the NR treatment. The observed trend again confirmed the N utilization mechanism. Ammonium is the top priority N source in diatom cells. Once ammonium is used up, the cells will assimilate extracellular nitrate and deoxidize it into nitrite through nitrate reductase, then pump the nitrite out of the cells^62^. In this process, most of the reduced nitrite is retained in the cell for further incorporation into biomass though some can leak out. After nitrate was exhausted, extracellular nitrite was assimilated into cells to maintain regular physiological metabolism on day 5. N deprivation has been demonstrated to have an impact on chlorophyll synthesis and even photosynthesis in many eukaryotic plants^63^. Likewise, when diatom cells were cultured in an N-deprived environment in the present study, the chlorophyll content decreased dramatically. The intracellular neutral lipid content increased when N deprivation occurred, which has been shown to occur in many other algae^31–37^.

### Processed RNA-Seq and proteome data

**Figure 2:**
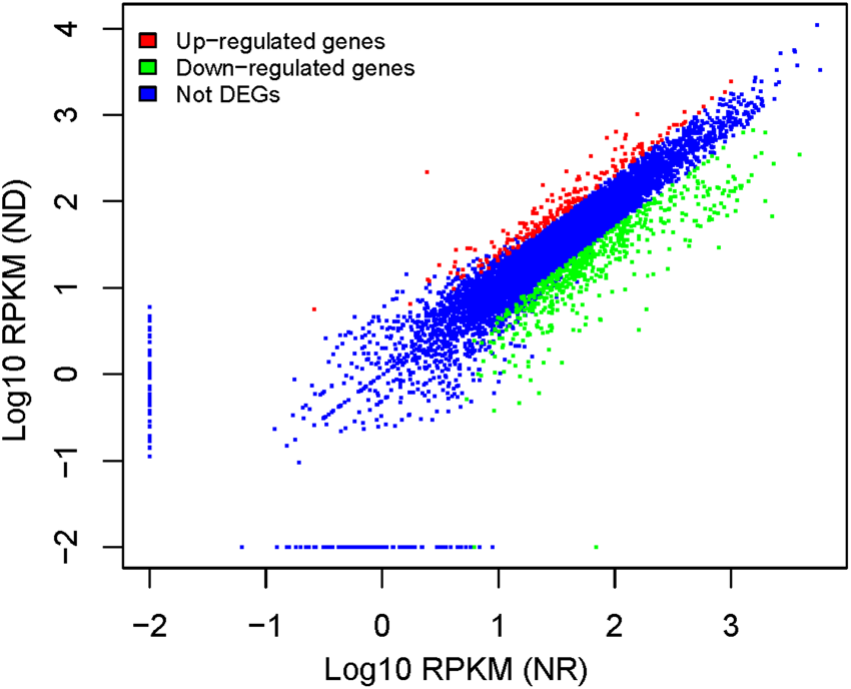
Differentially expressed genes under N repletion (NR) and N deprivation (ND). Distribution of gene expression (log10-transformed RPKM values (reads per kilobase per million mapped reads)) between NR and ND, with the differentially expressed genes highlighted. The colors represent different probability values. The darker regions represent a higher gene density.

To identify algal N-stress response-related genes in the diatoms, comparative transcript profiling of *T. pseudonana* cells during the mid-stationary phase (on the eighth day of culture) was performed under different N concentrations using RNA-Seq. A total of 6,264,454 and 6,249,215 high-quality reads (with the adaptors removed and low-quality data filtered out) were obtained, and approximately 64% and 42% of reads could be mapped to the genome or genes of the *T. pseudonana* genome in the ND and the NR conditions, respectively (Table S1). The expression level for each gene was determined from the numbers of reads that were uniquely mapped to the specific gene and the total number of uniquely mapped reads in the samples, which were calculated using the RPKM method (reads per kilobase per million mapped reads)^64^. The unique reads were mapped to 11,296 and 11,338 transcripts for ND and NR, respectively, which were clustered to a final consensus transcriptome of 11,385 transcripts (slightly larger than the reference gene model) using SOAP2.21. Based on the RNA-seq analysis, 11,241 transcripts were clustered to *T. pseudonana* CCMP 1335, and the remaining 145 transcripts were clustered to *Thalassiosira oceanica* (51), *T. pseudonana* (48, not the CCMP 1335 strain), *Phaeodactylum tricornutum* CCAP 1055/1(13), other species (13) and unidentified species (19) using the JGI database for user annotation references and sources for common gene names. A total of 782 differentially expressed genes (DEGs) were identified in this dataset. Two hundred and seven genes were up-regulated, and 575 genes were down-regulated in the ND treatment compared with the NR treatment (Fig. 2). The Blast2GO program was used to provide Gene Ontology (GO) annotation information. WEGO software^65^ was employed for GO functional classification for DEGs and to understand the overall distribution of gene functions in the species (Figure S2). The greatest numbers of DEGs among the three categories were the 250 terms obtained for metabolic processes (i.e., biological processes), the 174 terms for cell parts (i.e., cellular components) and the 211 terms for catalytic activity (i.e., molecular functions). Through KEGG pathway classification, 320 DEGs were annotated and assigned to at least one of the 86 metabolic pathways. The top 10 categories of pathways were as follows: metabolic pathways (135), biosynthesis of secondary metabolites (71), purine metabolism (24), glycolysis/gluconeogenesis (19), photosynthesis-antenna proteins (18), RNA transporter proteins (15), photosynthesis (13), carbon fixation in photosynthetic organisms (12) and amino sugar as well as nucleotide sugar metabolism (12) (Figure S3). The proteomics profile analysis was based on iTRAQ labels and protein extraction, in parallel with transcriptome profiling. From a total of 8,206 peptides, 8,093 unique peptides and 2,564 proteins were identified in the proteome. The mass of the identified proteins was mainly distributed between 20~50 kDa, and the highest percentage was ~15% in the 30~40 kDa range. The quantitative protein ratios were weighted and normalized based on the median ratio from Mascot2.3.02.The protein quantification and statistical significance of the protein quantitative ratios was performed by the software of Iquant, the candidate differentially expressed proteins ratios was identified with p-value < 0.05 and only fold changes >1.5, then the differentially expressed proteins (DEPs) were identified, among which 152 proteins were more abundant in the ND condition, while 210 proteins were more abundant in the NR condition. Among the total 362 DEPs, 196, 143 and 200 terms were classified as biological processes, cellular components and molecular functions, respectively. In the KEGG annotation analysis, 5 pathways showed at least 10 terms, including metabolic pathways (87), biosynthesis of secondary metabolites (47), photosynthesis (11), oxidative phosphorylation (11) and protein processing in the endoplasmic reticulum (10).

Integrated analysis of the transcriptome and proteome led to a total of 1,665 correlations being identified between quantitative transcripts and proteins as well as 80 correlations identified in both DEGs and DEPs. A further analysis showed a rather weak correlation between the protein and gene expression levels, with r = 0.1617. This phenomenon also appears in bacteria, and transcript abundance and protein abundances are not necessarily correlated^66^. On the other hand, the different datasets (i.e., transcript and protein) may provide key aspects of the physiological state in the cells. Previous studies on *P. tricornutum* based on several gene RT-PCR and related protein analyses showed similar abundance trends^67^. Based on whole-genome transcript and protein analyses conducted in this study and in *P. tricornutum*, some genes may show similar transcript and protein trends, but these trends are not correlated well with each other^60^. However, the different transcript and protein data demonstrated the same expression trends in 64 proteins, including 13 down-regulated and 51 up-regulated proteins, and a similar trend was found for the correlation coefficient was well (r = 0.5053). These results may further show that this approach may be more comprehensive for assessing genome-wide changes and the complex relationships between the transcripts and proteins.

### Transcripts of photosynthesis and carbon-fixation-related genes decreased

**Figure 3:**
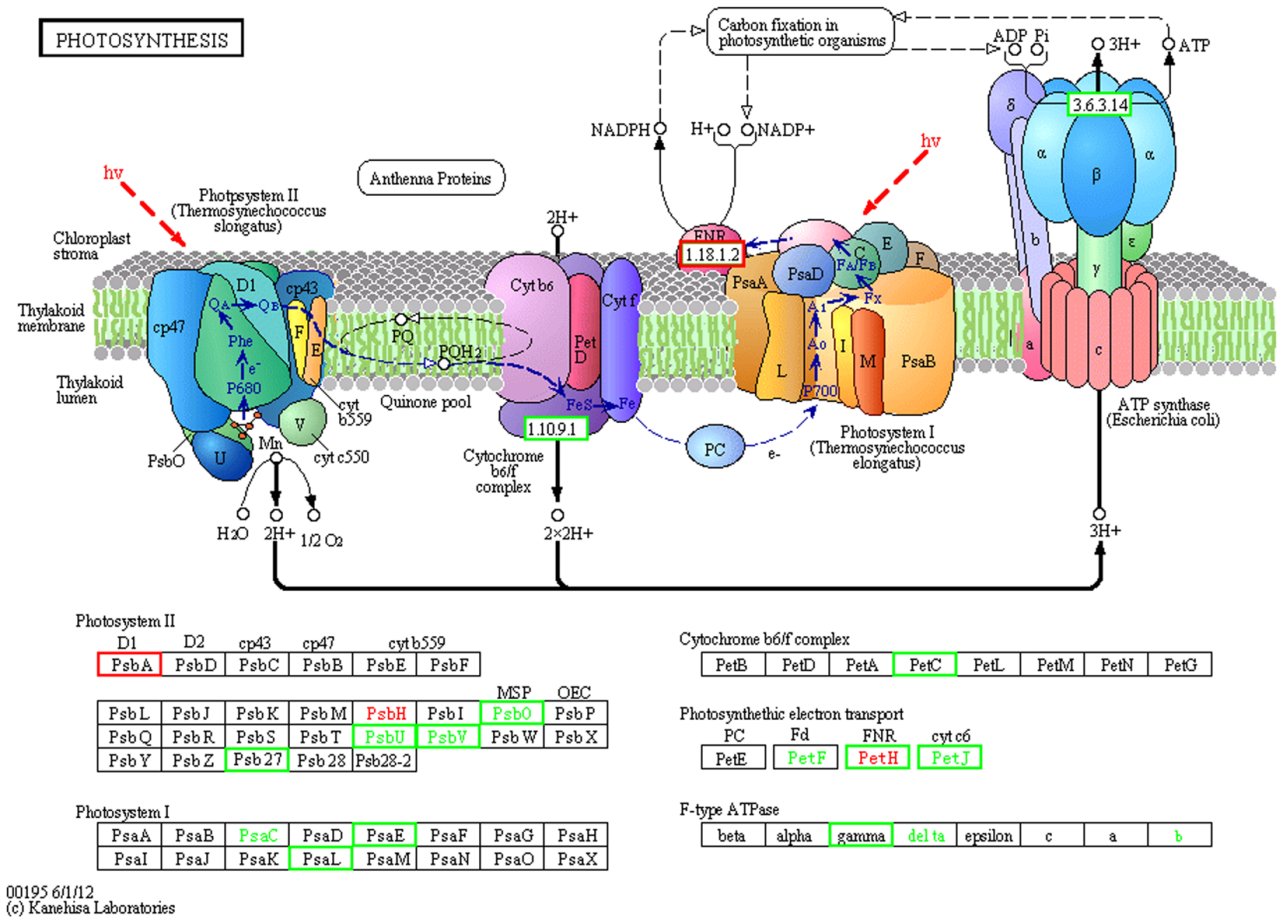
The KEGG photosynthesis pathway map for *T. pseudonana* during N depletion compared to N replete conditions (ND vs. NR). The KEGG photosynthesis pathway map can be found online at http://www.kegg.jp/pathway/map00195. The light green boxes indicate the genes were down-regulated and up-expressed (red boxes). The highlighted in red indicate the protein represent up-expressed and down-expressed (green word). The detail fold-change and annotation of these genes were summarized in Table 1 and Dataset 1.

N deprivation may influence photosynthetic efficiency in *T. pseudonana*, as the shown in Fig. 3 ref. 68 (detailed number as Table 1 and Dataset 1). The abundance of transcripts encoding photosynthesis-related proteins was substantially reduced, including those involved in chlorophyll biosynthesis and the efficiency of PSI and PSII. A similar phenomenon was discovered in *P. tricornutum* during N deprivation^69^. Chlorophyll is a nitrogenous macromolecule, and reduction of chlorophyll suppresses the nitrogen demand, in addition to affecting the light-harvesting capacity and production of reactive oxygen species^3^. In this study, the transcript encoding GSA markedly decreased by more than 10-fold. Chlorophyll b reductase, which is an enzyme that catalyzes Chl *b* to 7-hydroxy-chlorophyll *a*, also showed a reduction in both the transcriptomic and proteomic datasets. The abundance of transcripts encoding chlorophyll biosynthesis proteins was reduced for both the precursor and key enzymes, and this situation could decrease the chlorophyll content directly, which was consistent with the chlorophyll content indicated above. Most of the genes encoding antenna proteins in PS I and PSII were sharply down-regulated, especially in PS I, and the synthesis of chlorophylls and antenna proteins was inhibited (Dataset 1 and Fig. 3)^68^. This inhibition may have resulted in excess light energy. To protect itself from light injury, light-harvesting complex II could alter its function from light-harvesting to light-dissipating to reduce excess light energy^69^. Among all transcripts encoding antenna proteins, *PsbA* in the D1 protein was the only gene that was up-regulated 2-fold, which suggests that turn-over of the D1 protein became faster to prevent photo-inhibition(Fig. 3)^68, 70^. In diatoms, the xanthophyll cycle can induce non-photochemical quenching (NPQ) to protect PS II from excess light energy, and a decrease of NPQ therefore has a positive impact and harms PS II^71–73^. In the photosynthetic electron transport pathway, the reaction in the cytochrome *b*6 subunit of the *b*_*6*_ *f* complex is a rate-limiting step, and plastoquinone is the direct electron donor to cytochrome b6f ^74, 75^. In chloroplasts, dynamic ATP biosynthesis is hypothesized to occur through an ion gradient generated via electron transport^74^. For the PsbO of photosynthesis proteins and the *b*_*6*_ subunit of the cytochrome *b*_*6*_ *f* complex, both the transcripts and proteins were down-regulated (Fig. 3)^68^.

**Table 1:** The Fold Change of transcrpits and proteins in *T. pseudonana* under N deprivation.

	Protein Name	Gene ID	mRNA Fold Change		Protein Fold Change
**Photosynthesis and photosynthetic electron transport**					
1	magnesium chelatase	Thaps32201		−4.5	−2.9
2	chlorophyll b reductase	Thaps8063		−24.2	−1.8
3	PsbA (D_1_ Protein PSII)	Thaps1736		2	
4	PetC (PSI)	Thaps26131		−2.4	
5	PetH (FNR)	Thaps4914		−2.7	1.8
		Thaps25892		−8.1	
6	F-type ATPase	Thaps40156		−3.4	
**Calvin Cycle**					
7	Phosphoribulokinase	Thaps4376		−7.1	
8	Phosphoglycerate kinase	Thaps35712		−49.7	−1.6
9	Triosephosphateisomerase (TIM)	Thaps36462		−4.4	
10	Transketolase	Thaps21175		−4.8	
		Thaps26678			1.6
**Gluconeogenesis**					
11	Pyruvate carboxylase	Thaps269908		−3.2	
12	Fructose-1,6-bisphosphatase I	Thaps3302		−5.8	
**Starch biosynthesis**					
13	Phosphoglucomutase	Thaps35878		−22.2	
		Thaps268621		−5.6	
**Glycolysis**					
14	glucose-6P isomerase	Thaps38266		2.2	2
15	6-phosphofructo kinase	Thaps31232		2.9	
16	phosphogluceratemutase	Thaps27850		2.7	1.6
17	pyruvate kinase	Thaps264583		−2.1	
		Thaps22345			1.7
**Pentose phosphate pathway**					
18	ribose-5P isomerate	Thaps32332		−4.6	
19	ribulose-phosphate 3-epimerase	Thaps32924		−12.5	
20	Transketolase	Thaps21175		−4.8	
		Thaps26678			1.6
**TCA cycle**					
21	pyruvate dehydrogenase component (E_1_)	Thaps8778		2	
		Thaps268374		2.1	
22	dihydrolipoamideacetyltransferase (E_2_)	Thaps547		−36.8	
		Thaps21177			−1.5
23	dihydrolipoamide dehydrogenase (E_3_)	Thaps24399		−3.7	
		Thaps268657			1.5
24	pyruvate carboxylase	Thaps269908		−3.2	
25	citrate synthase	Thaps11411		2.4	1.9
26	isocitrate dehydrogenase	Thaps21640		2.7	1.6
**Oxidative phosphorylation**					
27	NADH dehydrogenase	Thaps262796		−5.3	
		Thaps38312		−2.1	
		Thaps23249			1.6
		Thaps38261			1.7
28	inorganic pyrophosphatase	Thaps269348		−6916.1	1.7
		Thaps5188		−5.8

As one part of the electron transport, NADPH was produced in the last step of the electron chain of the light reactions of photosynthesis. The NADPH-catalyzing enzyme ferredoxin-NADP^+^ reductase (the fold-change of Thaps25892 and Thaps4914 respectively is 6.0 and 2.8) showed a marked decrease in transcript abundance, but an increase in protein abundance. NADPH, pumped-protons and ATP were used to help turn the carbon dioxide into glucose. When the protons travel through the CF1 structure in F-type ATPase, the energy that is released causes the γ subunit to revolve to induce a conformational change and produce ATP^74, 76^. Therefore, the oxygen, NADPH and ATP generated through photosynthesis would all decrease, which finally regulated the carbon fixation.

**Figure 4:**
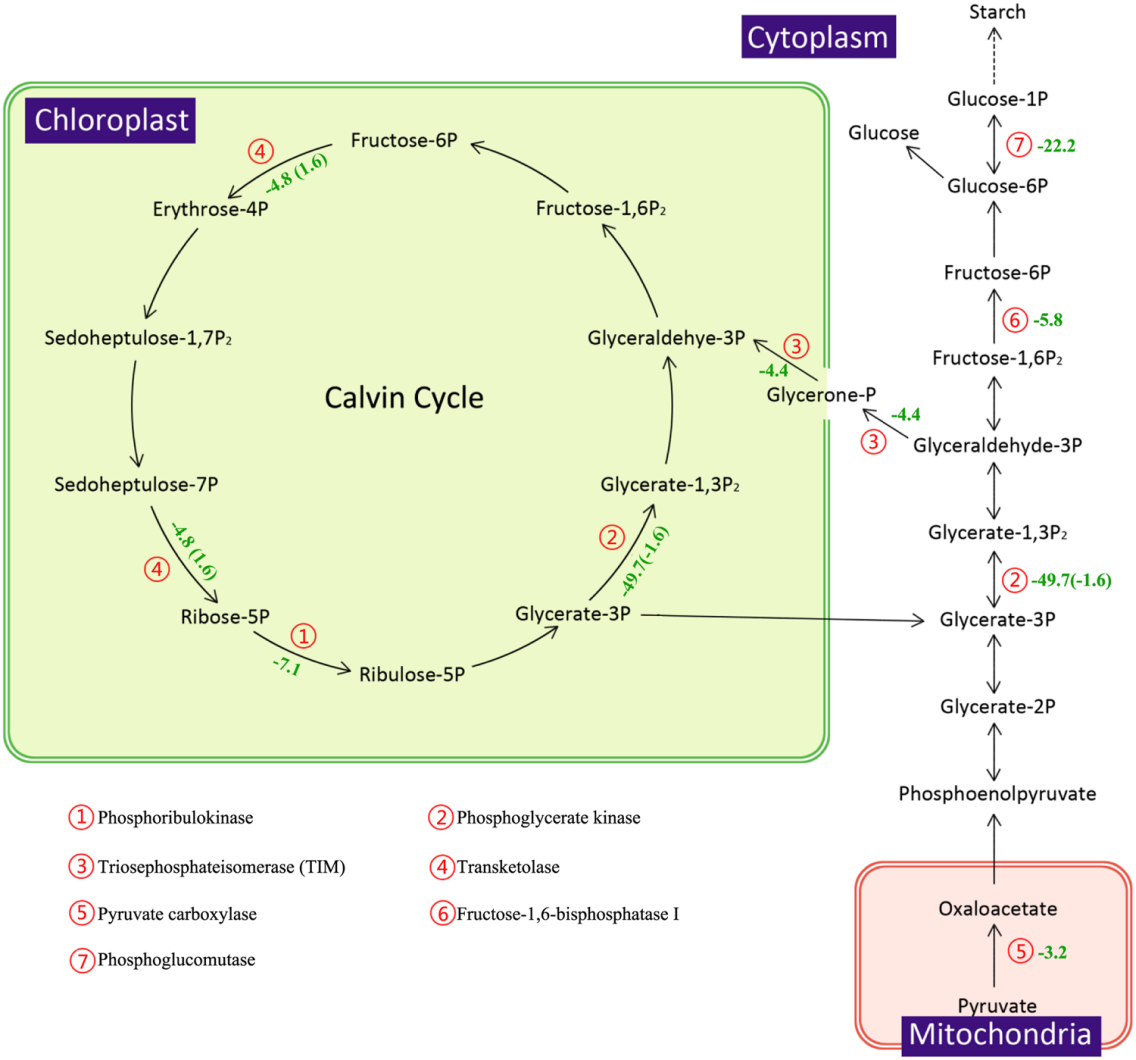
The KEGG calvin cycle and starch synthesis pathway for *T. pseudonana* during N depletion compared to N replete conditions (ND vs. NR). The serial number represent the related enzyme and the negative number indicate the down-expressed fold change of the genes (The opposite is up-expressed). The Inside the parentheses is the protein fold change.

Following N deprivation, the mRNA abundance of most enzymes in the Calvin cycle decreased, leading to a decline of the carbon fixation in *T. pseudonana* (Fig. 4). Among these genes, two ATP-dependent enzymes, phosphoribulokinase and phosphoglycerate kinase, showed dramatic decreases of 7.1 and 49.7-fold, which demonstrated that ATP biosynthesis had decreased in the photophosphorylation pathway. Due to *PEPCKase* and *PEPCase* genes of the *T. pseudonana* genome^10^, there is still no agreement whether *T. pseudonana* contains the C4 pathway^77, 78^. In this study, the abundance of PEPCase increased by 50% of the protein level observed in cells in f/2 medium, although we did not perform experiments assessing CO_2_ concentrations. Two key enzymes involved in gluconeogenesis, pyruvate carboxylase and fructose-1,6-bisphosphatase I decreased by 3.2 and 5.8-fold in terms of transcript abundance (Fig. 4). This regulation extended to the expression of the phosphoglucomutase gene, which catalyzes the transformation of glucose-6P to glucose-1P and finally suppresses starch synthesis. Thus, carbohydrate biosynthesis had been suppressed obviously in cells in an N-limited environment.

### Carbohydrate decomposition and utilization

**Figure 5:**
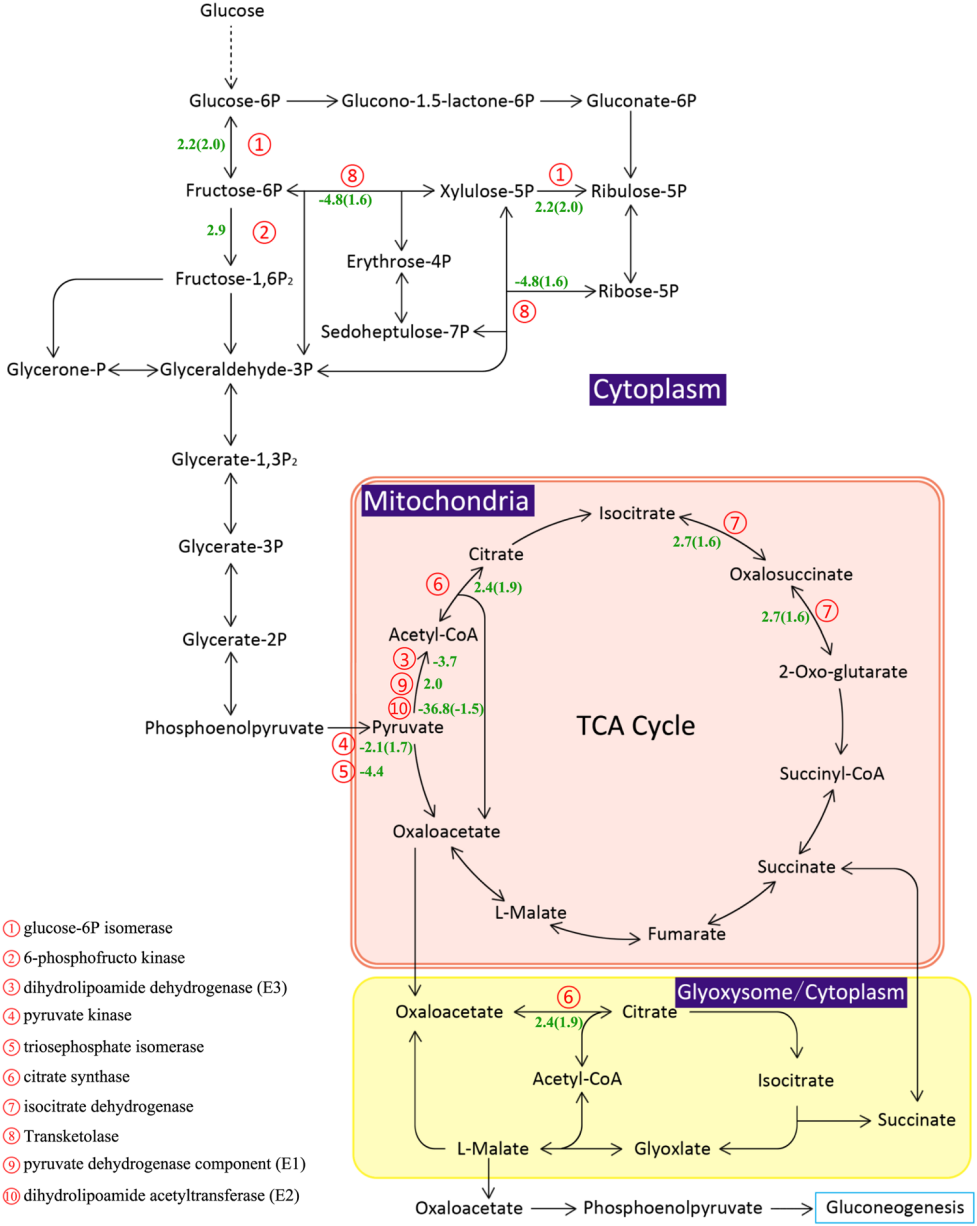
The glycolysis, pentose phosphate, TCA cycle and oxidative phosphorylation pathway in *T. pseudonana* during N depletion compared to N replete conditions (ND vs. NR). The serial number represent the related enzyme and the negative number indicate the down-expressed fold change of the genes (The opposite is up-expressed). The Inside the parentheses is the protein fold change.

As the key and rate-limiting enzyme in glycolysis, 6-phosphofructokinase transcript abundance was increased 2.9-fold (Fig. 5), which could be activated by AMP and suppressed by ATP. In addition, another key enzyme, pyruvate kinase, displayed a slight decrease in transcript abundance but showed the opposite trend in protein abundance (Fig. 5). The activity of this enzyme has an important influence on fatty acid biosynthesis^79^. These two enzymes are specific to glycolysis, indicating that this was the direction of carbon flow for producing more ATP and pyruvate. Pyruvate can be converted to acetyl-CoA as a carbon input for the TCA cycle.

The pentose phosphate pathway is another main pathway for degrading glucose and producing NADPH in cells; this pathway consists of an oxidative phase and a non-oxidative phase. However, the abundance of transcripts encoding ribulose-phosphate 3-epimerase and transketolase, which catalyzes the transformation of ribulose-5P to xylulose-5P and sedoheptulose-7P, and then to fructose-6P and glyceraldehyde-3P, both of which were decreased more than 5-fold. Ribose 5-phosphate isomerase, which catalyzes the transformation of ribulose-5P to ribose-5P (precursors of nucleotides), showed a 4.6-fold decrease (Table 1). To maintain regular metabolism during N deprivation, glucose preferentially enters glycolysis to produce ATP, rather than the pentose phosphate pathway for producing NADPH. Thus, as the main pathway for producing NADPH, the decrease in the phosphate pathway could affect fatty acid biosynthesis because fatty acid biosynthesis requires plentiful NADPH.

Pyruvate, as a carbon input into the TCA cycle, is converted to acetyl-CoA by the pyruvate dehydrogenase complex, which consists of pyruvate dehydrogenase (E_1_), dihydrolipoamide acetyltransferase (E_2_) and dihydrolipoamide dehydrogenase (E_3_). Among these three enzymes, both transcripts encoding E_1_ were increased to 2-fold of the level in the NR treatment. The abundance of the transcript encoding E_2_ was sharply down-regulated by 36.8-fold, while the level of the E_2_ protein only decreased slightly (Fig. 5). For E_3_, there was one down-regulated transcript, and one up-regulated protein was detected. On the other hand, pyruvate can be catalyzed to oxaloacetate by pyruvate carboxylase, and one transcript was decreased to almost one-third of the amount in the NR treatment (Fig. 5). Thus, how is the acetyl-CoA content altered in cells? The acetyl-CoA content shows an important relationship with fatty acid biosynthesis. Does the increased level of acetyl-CoA suppress the activity of E_2_ via negative feedback? From fatty acid biosynthesis and the degradation of the Val, Leu and Ile pathways, there was no signal for increasing acetyl-CoA. Additionally, acetyl-CoA is a strong activator for pyruvate carboxylase, which was found to be up-regulated in this study, indicating that the content of acetyl-CoA in cells was decreased (Fig. 5). Although the synthesis of acetyl-CoA was suppressed, the TCA cycle was enhanced due to two key enzymes, citrate synthase and isocitrate dehydrogenase, which were increased in both transcript and protein abundance. Additionally, the abundance of dihydrolipoamide succinyltransferase and the succinate dehydrogenase enzyme increased almost 2-fold. Enhancement of the TCA cycle typically indicates that an organism requires more ATP or intermediates as precursors for other pathways.

The oxaloacetate generated from the TCA cycle could be converted to phosphoenolpyruvate (PEP) and gluconeogenesis to produce glucose or serve as a carbon input into glyoxylate metabolism to form acetyl-CoA, which would in turn form oxaloacetate, which again participates in the TCA cycle. The biological function of the TCA cycle is to degrade triacylglycerol to acetyl-CoA and form glucose for cells. Glyoxylate metabolism showed not change, which was consistent with the decrease in gluconeogenesis.

Oxidative phosphorylation re-oxidizes NADH and FADH2, which are mainly generated from glycolysis and the TCA cycle through the electron transport pathway. However, both the transcriptome and proteome datasets showed decreases in NADH dehydrogenase.

### N utilization and metabolism

In the ND treatment, inorganic N was exhausted on day 4. Both the transcript and protein abundance of nitrate reductase and ferredoxin nitrite reductase, which catalyze nitrate to nitrite and nitrite to NH_3_, respectively, declined. After day 4, the nitrogen was not enough, the cellular chlorophyll content significantly declined and the activity of the nitrate reductase and ferredoxin nitrite reductase were affected. Therefore, the cells may produce stress reaction and showed an increased affinity for nitrogen, resulting in the increase of the reduced flavodoxin abundance.

There were changes in two glutamine synthetases responsible for catalyzing the transformation glutamate and NH_3_ to glutamine. One was decreased in mRNA and protein abundance, while the other one only showed changes in protein abundance, with an almost 2-fold increase being observed.

In amino acid metabolism pathways, both the transcriptome and proteome datasets showed that catabolism was faster than anabolism. Glutamate synthase catalyzes the transformation of glutamine to glutamate, which is then dehydrogenized to form 2-oxoglutarate; phenylalanine is catalyzed to form tyrosine and then fumarate; aspartate is catalyzed to form transaminase; lysine, leucine, valine, isoleucine, cysteine, alanine, glycine and serine are catalyzed to form acetyl-CoA; and these products are finally converted in the TCA cycle.

Following N depletion, citrate synthase and isocitrate dehydrogenase were up-regulated by 2.4 (transcription level), 1.9 (protein level) and 2.7 (transcription level), 1.6-fold (protein level), respectively (Fig. 5). The cells may decompose less important proteins more quickly, and the free amino acids may be used to synthesize new proteins or enter the TCA cycle to produce intermediates for other pathways in terms of maintaining regular catabolism.

### Lipid biosynthesis and metabolism

**Figure 6:**
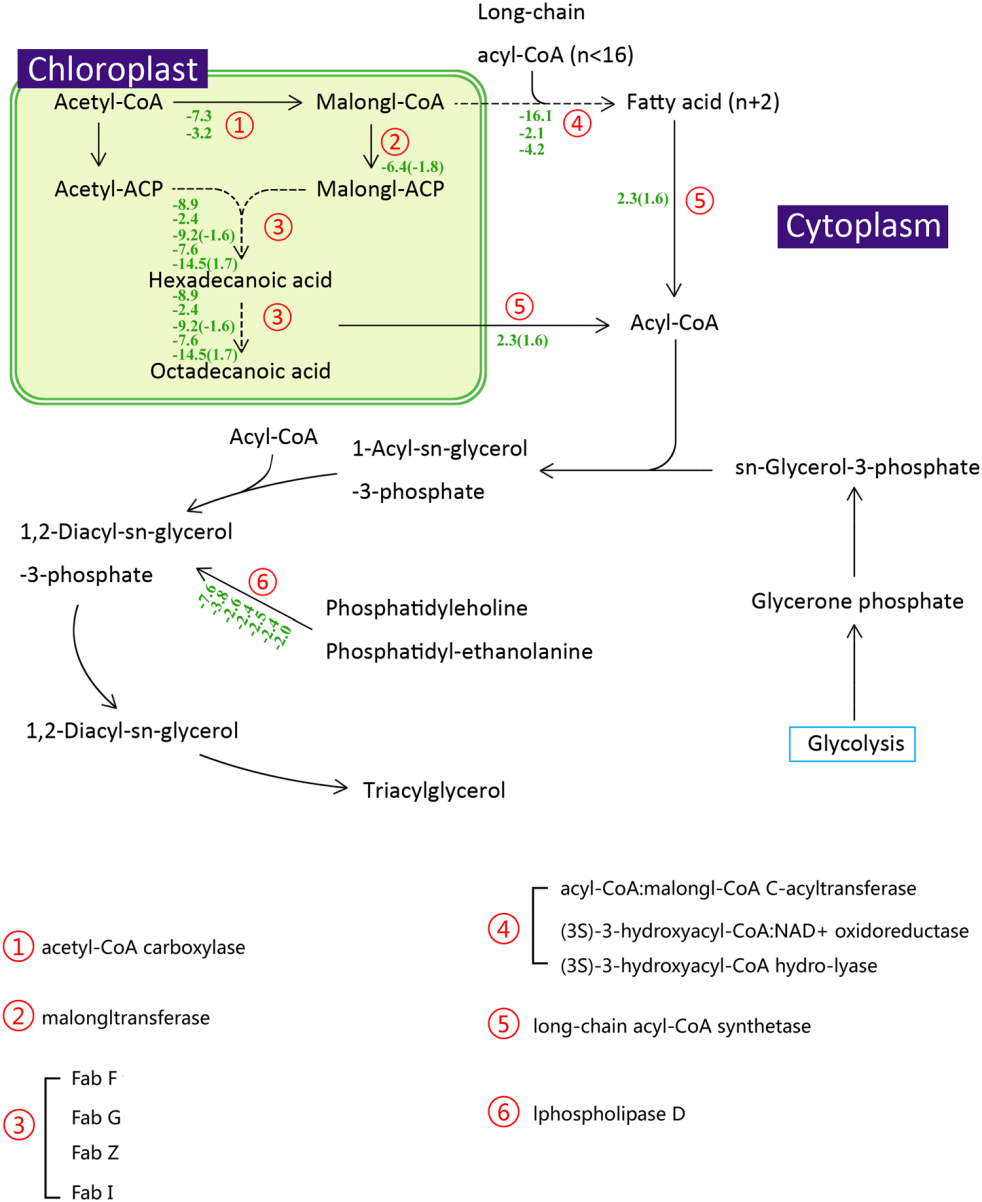
The lipid synthesis and metabolism pathway in *T. pseudonana* during N depletion compared to N replete conditions (ND vs. NR). The serial number represent the related enzyme and the negative number indicate the down-expressed fold change of the genes (The opposite is up-expressed). The Inside the parentheses is the protein fold change. There are serval genes in one enzyme.

Fatty acid *de novo* biosynthesis was found to be suppressed in both the transcriptome and proteome datasets, as the key enzyme acetyl-CoA carboxylase showed a decrease in transcript abundance. Acetyl-CoA carboxylase (ACCase), which catalyzes the transformation of acetyl-CoA into malonyl-CoA (an important precursor in the synthesis of fatty acids), decreased dramatically, as did other enzymes in this pathway (Fig. 6). Although ACCase is regarded as a key enzyme in fatty acid biosynthesis, overexpression of ACCase in diatoms does not improve the intracellular lipid content^80^. As a decrease in fatty acid biosynthesis occurs, palmitic acid elongation is also suppressed, including elongation in mitochondria and on the endoplasmic reticulum (Fig. 6). The restriction of fatty acid biosynthesis could be due to the decrease in acetyl-CoA contents mentioned above as well as the decrease in NADPH, which is treated as the reducing power in fatty acid biosynthesis and is mainly generated through the pentose phosphate pathway. This reduction could also be the reason that ACCase overexpression occurred without any increase in lipid content (Fig. 6).

In this study, there was no significant evidence that the fatty acid β-oxidation pathway became more active. Only long-chain acyl-CoA synthetase, which catalyzes long-chain fatty acids to acyl-CoA, showed a marked increase in both mRNA and protein abundance. Following that increase, acyl-CoA could have been transported into mitochondria through the synergistic effects of carnitine acyltransferase I and carnitine/acylcarnitine translocase and subsequently entered the β-oxidation pathway. In addition, acyl-CoA was is of the sources for TAG synthesis.

According to the Nile Red data, the intracellular lipid content (mainly consisting of TAG) was increased under the ND treatment. However, neither the transcriptome nor proteome dataset showed any enhancement of enzymes related to TAG biosynthesis, including cytosolic glycerol-3-phosphate acyltransferase (GPAT), lysophosphatidic acid acyltransferase (LPAAT), phosphatidic acid phosphatase (PAP) and diacylglycerol acyltransferase (DGAT). This finding is similar with previous studies. Hockin *et al*. did not detect any obvious changes in the enzymes involved in the fatty acid and lipid biosynthesis pathways via protein analysis^81^. The abundance of another source of TAG, sn-glycerol-3-phosphate, which is transformed from glycerone phosphate, also showed no change.

Hence, what was the source of TAG? In *Chlamydomonas reinhardtii*, TAG is assumed to be remodeled from membrane phospholipids^38^. Phospholipid:diacylglycerol acyltransferase (PDAT) can catalyze the transformation of phospholipids to diacylglycerol (DAG) as a precursor to TAG synthesis^82, 83^. In the present study, there was no signal of any changes in PDAT in the transcriptome or the proteome. Interestingly, phospholipase D can carry out the catalysis of phosphatidylcholine and phosphatidyl-ethanolamine (both of which are major components in membrane phospholipids) to 1,2-diacyl-sn-glycerol-3-phosphate, and the transcript abundance of this enzyme showed a decrease in the present study. All of these findings indicated that phospholipid degradation was suppressed in *T. pseudonana*, which was quite different from what occurs in green algae^38, 45^. However, our results do not allow us to deduce the original source of TAG. Thus, additional experiments will be required to explore this unanswered question.

## Conclusion

In this study, *T. pseudonana* displayed a significant decrease in photosynthesis, including decreases in chlorophyll and antenna protein biosynthesis, electron transport and photophosphorylation, in both RNA-seq and proteome data, which further resulted in decreased carbon fixation and gluconeogenesis abilities under N depletion. The enhancement of glycolysis and the TCA cycle and the maintenance of the pentose phosphate pathway and the glyoxylate cycle indicated that cells preferred to preserve the functions of the main pathways to generate energy and intermediates. Furthermore, oxidative phosphorylation was decreased in the cells to slow their metabolism, with only intermediates for the most required substances being catalyzed, thereby resisting negative external impacts. As N became exhausted in the media, cells began to degrade unnecessary proteins to produce free amino acids to synthesize more important proteins or to enter the TCA cycle to maintain their metabolism. Although the intensity of Nile Red fluorescence showed a marked increase under the ND treatment compared with that under the NR treatment, we could not find any evidence of enhancement of transcripts or proteins involved in TAG biosynthesis pathways. There may be some undetected pathways or mechanisms that produce TAG, and we will need to conduct further experiments to evaluate this possibility. A combination of RNA and protein profiling was shown to be able to reveal most of the genes in pathways associated with physiological activity under nitrogen-depleted conditions in *Thalassiosira pseudonana*, which could be valuable for future physiological research in phytoplankton.

## Electronic supplementary material

Dataset 1

The Combination of RNA and Protein Profiling Reveals the Response to Nitrogen Depletion in Thalassiosira pseudonana
