# Supplementary material for: The Combination of RNA and Protein Profiling Reveals the Response to Nitrogen Depletion in Thalassiosira pseudonana
Source: Sci Rep. 2017 Aug 21;7:8989. doi: 10.1038/s41598-017-09546-x (PMC5566445; doi:10.1038/s41598-017-09546-x)
Supplement: Supplementary file 2 — The Combination of RNA and Protein Profiling Reveals the Response to Nitrogen Depletion in Thalassiosira pseudonana [file 41598_2017_9546_MOESM2_ESM.pdf]

The Combination of RNA and Protein Profiling Reveals the Response to Nitrogen Depletion in *Thalassiosira pseudonana*

Jianbo Jian[a], Dezhi Zeng[a], Wei Wei[a], Hongmin Lin[a], Ping Li[a*], Wenhua Liu[a*]

[a] Marine Biology Institute, Shantou University, Shantou, Guangdong 515063, P.R. China

[*] **Correspondence to: Liu Wenhua (PhD)**

Marine Biology Institute

Shantou University

Shantou, Guangdong 515063,

P.R. China

Tel.: +86-754-86503269

E-mail: whliu@stu.edu.cn

Li Ping (PhD)

Marine Biology Institute

Shantou University

Shantou, Guangdong 515063,

P.R. China

Tel.: +86-754-86503269

E-mail: liping@stu.edu.cn

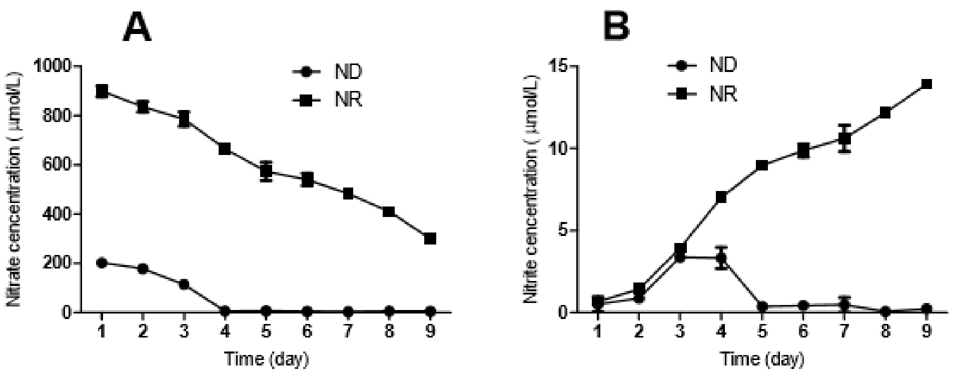

Figure S1. Detection of the N concentration in culture medium. (A) Nitrate concentration in the medium. (B) Nitrite concentration in the medium. Data are displayed as the means ± SD (n = 3).

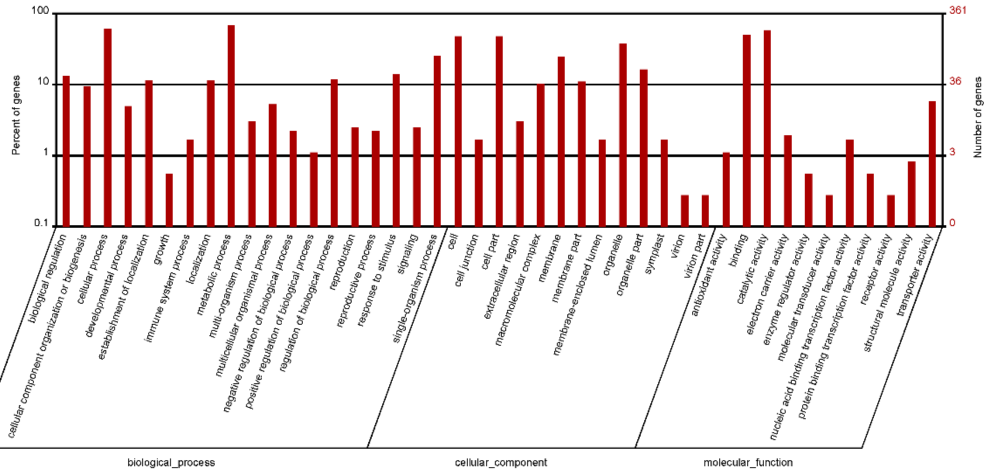

Figure S2. **Histogram presentation of the Gene Ontology classification for DEGs.** Three main categories are summarized for the significant DEGs: biological process, cellular component and molecular function. The right y-axis indicates the number of genes in a category. The left y-axis indicates the percentage of a specific category of genes in that main category.

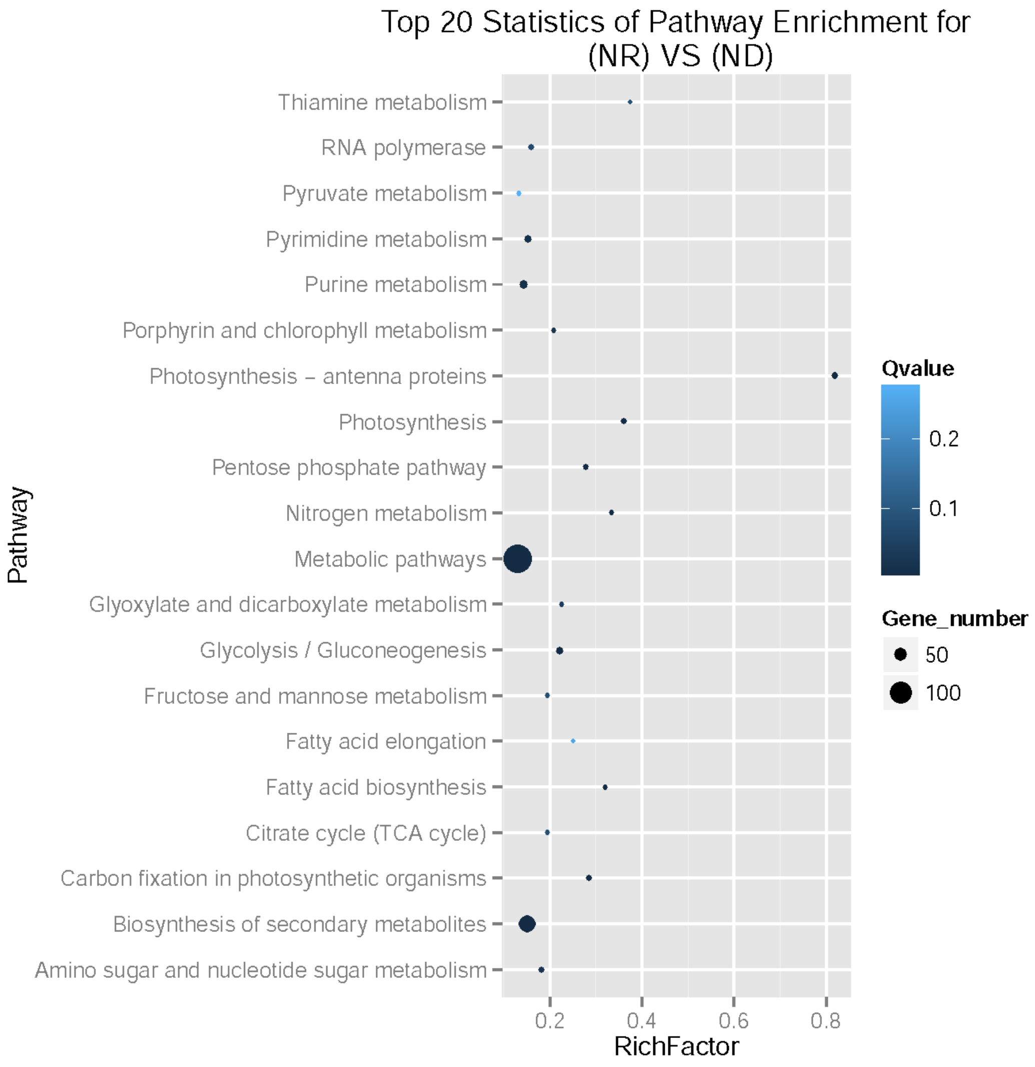

Figure S3. Scatterplot of the KEGG pathway enrichment statistics of DEGs between NR and ND. The RichFactor is the ratio of the number of differentially expressed genes annotated in that pathway to the number of all genes annotated to that pathway term. A larger RichFactor is associated with a higher intensity. The Q value is a corrected p-value ranging from 0~1, and a lower Q value is associated with a higher intensity. This figure only displays the top 20 pathway terms that were enriched.

Table S1. The N-depleted and N-replete reads were mapped to the *T. pseudonana* genome and genes.

	N depletion		N repletion	
Map to Genome	Reads number	Percentage	Reads number	Percentage
Total Reads (a)	6,264,454	100.00%	6,249,215	100.00%
Total Base pairs (a)	306,958,246	100.00%	306,211,535	100.00%
Total Mapped Reads (b)	4,016,018	64.11%	4,054,120	64.87%
Perfect match (c)	3,232,684	51.60%	3,277,736	52.45%
<=3bp mismatch (d)	783,334	12.50%	776,384	12.42%
Unique match (e)	3,843,747	61.36%	3,868,505	61.90%
Multi-position match (f)	172,271	2.75%	185,615	2.97%
Total Unmapped Reads (g)	2,248,436	35.89%	2,195,095	35.13%
Map to Gene	Reads number	Percentage	Reads number	Percentage
Total Reads (a)	6,264,454	100.00%	6,249,215	100.00%
Total Base pairs (a)	306,958,246	100.00%	306,211,535	100.00%
Total Mapped Reads (b)	2,683,085	42.83%	2,669,117	42.71%
Perfect match (c)	2,190,662	34.97%	2,192,715	35.09%
<=2bp mismatch (d)	492,423	7.86%	476,402	7.62%
Unique match (e)	2,572,610	41.07%	2,551,580	40.83%
Multi-position match (f)	110,475	1.76%	117,537	1.88%
Total Unmapped Reads (g)	3,581,369	57.17%	3,580,098	57.29%

Dataset 1. Statistics for photosynthesis- and carbon fixation-related transcripts. The results are shown for the NR and ND treatments, including expression, RPKM, FDR and annotation data (KEGG Orthology, GO Component, Function and Process).
